# Prevalence of culicine salivary antibodies in non‐human primates living in national parks in Thailand

**DOI:** 10.1111/mve.12779

**Published:** 2024-11-25

**Authors:** Ariza Minelle A. Aguila, Kobporn Boonnak, Daraka Tongthainan, Onrapak Reamtong, Sarocha Suthisawat, Oranit Likhit, Wirasak Fungfuang, Jeffrey Hii, Patchara Sriwichai

**Affiliations:** ^1^ Department of Medical Entomology, Faculty of Tropical Medicine Mahidol University Bangkok Thailand; ^2^ Department of Immunology, Faculty of Medicine Siriraj Hospital Mahidol University Bangkok Thailand; ^3^ Faculty of Veterinary Medicine Rajamangala University of Technology Tawan‐ok Chonburi Thailand; ^4^ Department of Molecular Tropical Medicine and Genetics, Faculty of Tropical Medicine Mahidol University Bangkok Thailand; ^5^ Department of Zoology, Faculty of Science Kasetsart University Bangkok Thailand; ^6^ College of Public Health, Medical and Veterinary Sciences James Cook University North Queensland Queensland Australia; ^7^ Present address: Research Institute for Tropical Medicine Muntinlupa Philippines

**Keywords:** *Aedes aegypti*, *Aedes albopictus*, *Culex quinquefasciatus*, macaques, mosquito bites

## Abstract

Macaques are widely distributed in Thailand with remarkable adaptation to living close to humans in residential, religious sites, markets and tourist areas. They play an essential role in the persistence of pathogens in the environment. As reservoir hosts, they are exposed to hematophagous vectors that secrete saliva, a cocktail of bioactive molecules including antigenic components stimulating host antibody production. Subsequent to the detection of mosquito‐borne pathogens in macaques living in national parks, we aimed to determine the seroprevalence of antibodies to crude salivary gland extracts (SGEs) from culicine mosquitoes (*Aedes aegypti* [Linnaeus, 1762], *Ae. albopictus* [Skuse, 1895] and *Culex quinquefasciatus* [Say, 1823]) and compare individual titres between macaque species/national parks (33, *Macaca arctoides* [I. Geoffroy Saint‐Hilaire, 1831] [Primates: Cercopithecidae] from Kaeng Krachan, 23 *M. leonina leonina* [Blyth, 1863] [Primates: Cercopithecidae] from Khao Yai and four *M. fascicularis* [Raffles, 1821] [Primates: Cercopithecidae] from Mu Ko Ranong). The anti‐mosquito SGE antibodies found in 60 macaques from three national parks indicate varying levels of host‐vector exposure. Macaque antibody titres were high against culicine mosquitoes. However, the significant difference among national parks (or macaque species) was only observed against *Cx. quinquefasciatus.* Correlation analysis of titres between *Aedes* SGE and arboviruses revealed a significantly more intense immune response against *Ae. albopictus* in DENV3‐positive *M. arctoides.* Current findings support the concept of salivary biomarkers using accessible SGE, available from mosquito colonies of interest*.* However, we observed cross‐reactivity between *Aedes* species because of crude SGE containing species‐shared proteins. Nevertheless, a potential risk of pathogen transmission is emphasised between national park visitors and macaques via mosquitoes as bridge vectors. This information contributes to preventive measures against mosquito bites, including those implemented in tourist areas.

## Graphical abstract

- Anti‐mosquito salivary gland antibodies were detected in the sera of macaques (*Macaca arctoides*, *M. leonina* and *M. fascicularis*) from Kaeng Krachan, Khao Yai and Mu Ko Ranong National Parks, respectively.
- For *M. leonina* and *M. fascicularis*, the highest titres were against *Ae. aegypti* while *Ae. albopictus* antibody titres were observed in *M. arctoides*.
- Cross‐reactivity was observed between the two *Aedes* species due to the use of crude salivary gland extracts, which may contain species‐shared proteins from the related *Aedes* (subgenus *Stegomyia*) species.

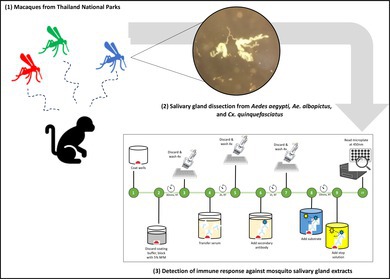

## INTRODUCTION

Arboviruses such as dengue (DENV), chikungunya (CHIKV) and Zika (ZIKV) originate in non‐human primates (NHPs). Arboreal mosquitoes transmit them from infected to naïve animals in a sylvatic transmission cycle and eventually spillover into the human cycle. The adaptation of NHPs to human habitations is likely to pose a more significant risk due to spillover transmission via bridge vectors, which are competent mosquitoes feeding on wild animals and humans. Human activities like encroachment on natural forest habitats for hunting, agriculture, deforestation, urbanisation, or tourism activities by people who are bitten by arbovirus‐carrying mosquitoes contribute to the risk (Han et al., 2019; Miot et al., 2020; Narat et al., 2017; World Health Organization, 2020). The resource‐sharing and spatial overlap of humans and macaques are evident in many areas in Thailand, such as neighbourhoods, religious sites, schools, business establishments, main roads, train tracks and marketplaces (Malaivijitnond et al., 2011). Their widespread distribution and the high prevalence of mosquito‐borne diseases highlight the importance of studying macaque interactions with mosquito vectors and their role as reservoir hosts in the sylvatic cycle.

Vector‐host contact is measured to understand the dynamics of pathogen transmission. It is achieved by determining vector density as a proxy estimate of mosquito bite rate on vertebrate hosts or analysing the collected mosquitoes' bloodmeal to identify its blood source. The animal‐baited trap is the reference for assessing mosquito biting rates on animals by tethering (water buffalo) or caging (monkey) animals inside a larger net, allowing mosquitoes to feed on them (Hawkes et al., 2017). Another technique, the monkey‐baited electrocuting net trap, utilises the monkey's odour piped from an enclosed tent opposite to an electrified grid to collect the attracted mosquitoes. However, surveillance of mosquitoes via electrocuting net traps showed low efficiency (Malijan et al., 2021). In addition to the trap's poor performance, other limitations include the possibility of escapees, animal welfare concerns due to restraining monkeys as baits in traps and challenges in setting up the traps (Service, 1993; Silver, 2008). Mosquito collection from other available trappings (e.g. light or carbon dioxide traps) is transported to the laboratory for blood meal analysis, which exploits the host blood left in the mosquito's abdomen after blood feeding. It directly identifies potential hosts of mosquitoes from a recent bloodmeal through available serological (haemoglobin crystallisation, passive hemagglutination inhibition and enzyme‐linked immunosorbent assays or [ELISA]) and molecular techniques (polymerase chain reaction and sequencing). Serological methods require easy‐to‐prepare reagents and simple equipment, and interpretation of immunoglobulin G (IgG) conjugated results is straightforward (Washino, 1983). Molecular approaches employ host gene fragment amplification using primers that amplify the DNA of a wide range of vertebrates. However, both of these techniques are limited by some factors such as cross‐reaction from closely related species (serological), availability of anti‐sera against target host species (serological), and enough DNA in the bloodmeal and reference libraries of gene sequences for the potential vertebrate hosts (molecular) (Gutiérrez‐López et al., 2022 ).

There is a burgeoning interest in using mosquito salivary proteins to probe species antibodies in human sera as a biomarker tool for assessing human exposure to mosquitoes and other blood‐sucking arthropods, the risk of mosquito‐borne disease transmission, and the efficacy of vector control measures (Sagna et al., 2018). The potential applications of salivary biomarkers to *Aedes*, *Anopheles* and *Culex* have been studied worldwide, especially in areas endemic for dengue or malaria in French Polynesia, Europe, North and South America and tropical Africa (Drame et al., 2010; Fontaine et al., 2011; Londono‐Renteria et al., 2010; Londono‐Renteria et al., 2020; Mathieu‐Daude et al., 2018). In Thailand, humoral immune responses to *Anopheles* and *Aedes* salivary proteins were evaluated for human‐exposure risk assessment to malaria and dengue, respectively (Fustec et al., 2021; Machain‐Williams et al., 2012; Waitayakul et al., 2006). The tool utilises mosquito saliva injected while probing for blood vessels. The saliva comprises a complex array of proteins, including antigenic components recognised by the host immune system and stimulating antibody production (Sagna et al., 2018). The salivary biomarker has been described to be highly sensitive, as evidenced by the heterogeneity of exposure and relative specificity, and capable of detecting both temporal and spatial variations (Hemme et al., 2016; Leitner et al., 2017; Poinsignon et al., 2010; Sagna et al., 2018). Moreover, the intensity of the response is proportional to the degree of exposure to mosquito bites, and a strong immune reaction to vector bites indicates a higher risk of transmission and infection (Mathieu‐Daude et al., 2018; Remoue et al., 2006; Sagna et al., 2018). In addition, salivary biomarkers have also been tested in vertebrate animal hosts such as chickens, equines, bovines and caprines (Boulanger et al., 2011; Trevejo & Reeves, 2005). Macaques living in Thailand national parks were found to be infected with dengue, Zika, malaria and Japanese encephalitis, indicating their circulation in the sylvatic cycle (Lakhotia et al., 2023; Nakgoi et al., 2014; Tongthainan et al., 2020). In this case, it is crucial to understand whether there is a potential spillover from vertebrate animals to human hosts by examining contact between the sylvatic host and local vectors. However, there is limited information on seroepidemiological studies of macaque‐mosquito interactions in areas endemic to *Aedes*‐borne diseases.

This cross‐sectional study aimed to investigate and characterise the seroprevalence of dominant national park‐dwelling macaque populations against mosquito salivary gland proteins. Crude salivary gland extract (SGE) from *Aedes aegypti* (Linnaeus, 1762), *Ae. albopictus* (Skuse, 1895) and *Cx. quinquefasciatus* (Say, 1823) was used to screen and monitor the macaque antibody response to mosquito exposure. The antibody titre profiles between macaque species/national parks and their correlation to arbovirus titres were assessed. The findings of this study suggest that macaque‐mosquito interactions in these areas may serve as a potential spillover pathway from vertebrate animals to human hosts.

## MATERIALS AND METHODS

### Macaque serum samples

**FIGURE 1:**
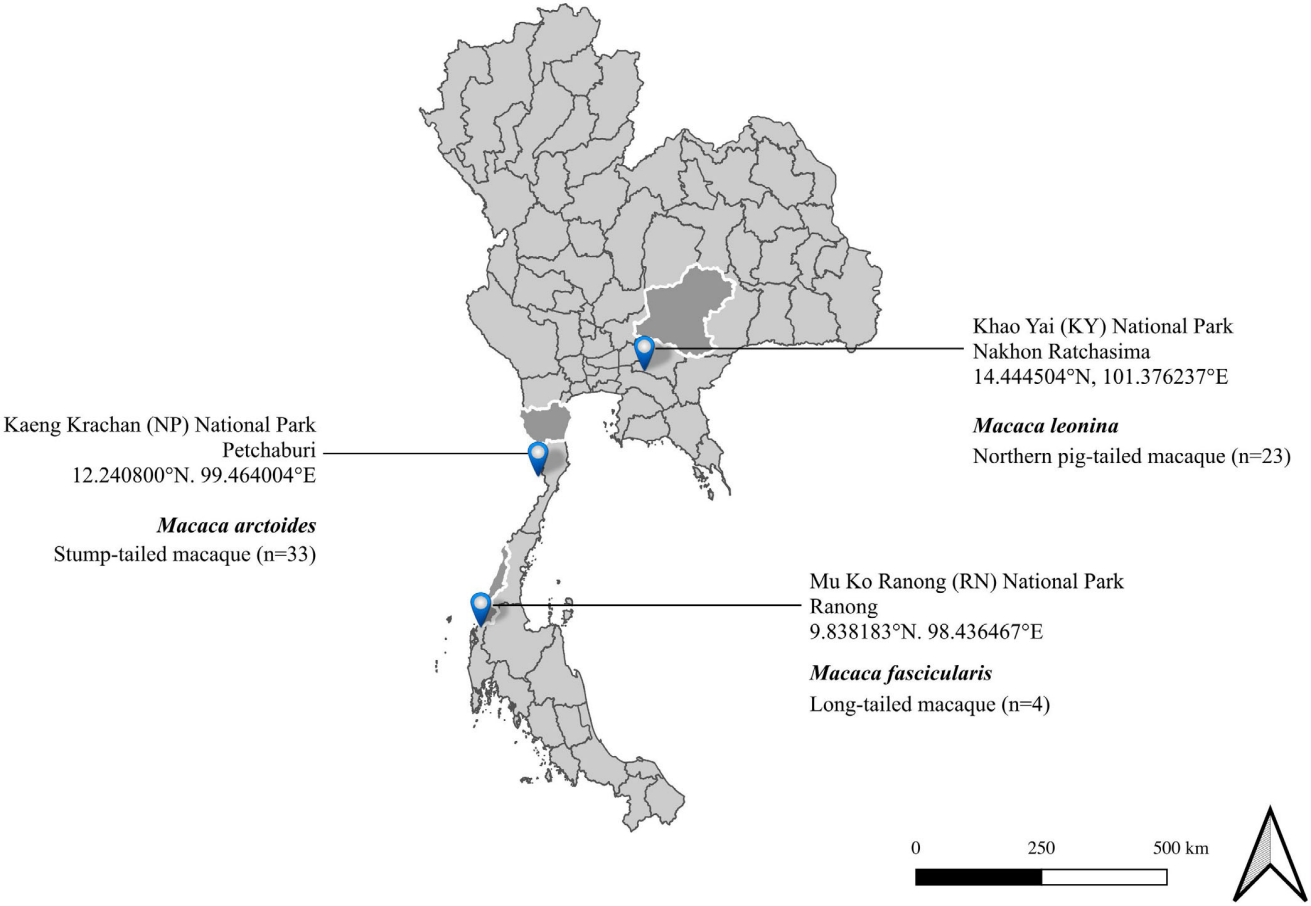
Map of serum samples collected from macaques in Thailand national parks from 2018 to 2019.

We used archived macaque serum samples (*n* = 60) initially obtained for an arbovirus seroprevalence study in 2018–2019 (Tongthainan et al., 2020). We aimed to survey three different species, and so we chose three national parks with a different macaque species as the most dominant according to their distributions that were reported previously (Aggimarangsee, 1992; Albert et al., 2013; Malaivijitnond & Yuzuru, 2008; Treesucon, 1988). This comprised 23 blood samples from wild *Macaca leonina* (northern pig‐tailed macaque) (Blyth, 1863) (Primates: Cercopithecidae) from Khao Yai National Park (KY), 33 samples from wild *M. arctoides* (stump‐tailed macaque) (I. Geoffroy Saint‐Hilaire, 1831) (Primates: Cercopithecidae) from Kaeng Krachan National Park (KK) and four samples from *M. fascicularis* (long‐tailed macaque) (Raffles, 1821) (Primates: Cercopithecidae) collected in Chang Island in the Andaman sea, Mu Ko Ranong National Park (RN) (Figure 1).

The monkeys were captured using a ground trap placed near a waterfall in KK and a feeding area in KY and RN. Macaque trapping for blood collection was previously described, wherein macaques were captured by ground trapping and sedated for blood collection by venipuncture of the inguinal vein. Sex and anthropological measurements (weight, arm length, leg length, tail length and body length) were taken from captured macaques, in addition to dental casts and photographs (Tongthainan et al., 2020). Sera from different sites were stored at −80°C.

KY is in Nakhon Ratchasima province, a 2168 km^2^ rainforest and grassland area home to the diverse wildlife of birds, reptiles and mammals, including the northern pig‐tailed macaque. Tourists can visit waterfalls accessible by car or guided on several trails. In KK, the Pa LaU waterfall and smaller caves can be found in the national park in the Petchaburi province. Artificial ponds near the campsites are filled with water during the rainy season for animal water sources. Human settlements are approximately 5 km away from the stump‐tailed macaque trapping points in KK. Meanwhile, long‐tailed macaque collection in RN is located in Ranong province covering land, coast and marine resources, including beaches, and evergreen and mangrove forests. Tourists can hike, camp, watch wildlife and visit waterfalls and viewpoints in RN Marine National Park (Thai National Parks, 2021). Generally, the wet and dry seasons fall from May to October and November to April, respectively.

### Dissection of mosquito salivary glands

Non‐blood‐fed 5‐day‐old laboratory‐strain female *Ae. aegypti* (Bora‐bora strain, Malaysia, F82 generation), *Ae. albopictus* (Rayong strain, Thailand, F33 generation) and *Cx. quinquefasciatus* (Khon Kaen strain, Thailand, F235 generation) were dissected to obtain the salivary glands. Mosquito colonies were reared at 27 ± 2°C, 65 ± 10% relative humidity and 12:12 light and dark cycle in the Department of Medical Entomology's physical containment level 2 insectary at Mahidol University's Faculty of Tropical Medicine. After chilling inside an ice chest box (~10°C), mosquitoes were immobilised entirely by removing the legs and wings for dissection of salivary glands on a glass slide with a drop of 1X phosphate‐buffered saline (PBS) under a stereomicroscope (Nikon SMZ 745T). The salivary gland pair was teased out using a dissecting probe after detaching the head from the thorax by gently pulling them in the opposite directions (Schmid et al., 2017). Dissected salivary glands were rinsed in another drop of 1X PBS before pooling into 100 pairs per batch (Coleman et al., 2007). The pooled salivary glands in PBS were manually homogenised using a micropestle for protein quantification. The protein content of the SGE antigen used to coat the wells was measured using the Bradford test (Kielkopf et al., 2020).

### Anti‐SGE antibody detection and titre determination

The anti‐SGE immunoglobulin G (IgG) antibody titre profile was determined by ELISA. Optimisation was performed through a checkerboard method to optimise the assay's serum and secondary antibody conditions. After optimisation of adjusting the antigen, serum and secondary antibody concentrations, 96‐well flat‐bottom microtitre plates (Immulon® Maxisorp, Nunc, Germany) were coated with 2 μg/mL SGEs prepared in coating solution at 50 μL/well (KPL, Gaithersburg, USA). The plates were sealed and incubated at 4°C overnight. We blocked the plates with a 200 μL blocking buffer of 1X PBS and 5% non‐fat dry milk for at least 30 min at room temperature. Serum samples, prepared at a two‐fold dilution series (1:200 to 1:204,800), were added to the plate‐bound antigen at room temperature for 120 minutes. Next, 50 μL/well of horseradish peroxidase‐labelled goat anti‐monkey IgG (Abcam, Cambridge, UK) diluted with 5% non‐fat dry milk at 1:1000 was incubated for 1 h at room temperature. A washing solution (1X + PBS‐Tween20) (Sigma‐Aldrich, St. Louis, USA) was used between reagents to remove unbound materials, and washing was repeated at least four times. Wells were incubated for 10 min at room temperature with 50 μL TMB Peroxidase Substrate System (KPL, Gaithersburg, USA), and enzyme activity was stopped with 50 μL sulfuric acid. An ELISA microplate reader measured absorbance or optical density (OD) at 450 nm/630 nm (Tecan, Switzerland).

### Data analysis

The adjusted optical density (∆OD) was calculated as the difference between the OD readings in the antigen‐containing wells (OD𝑥) and the blank well (OD𝑛) to determine the level of IgG immune response. From the ∆OD, the serum antibody titre was determined to be the maximum serum dilution that produced an (∆OD) value of 0.2. After verifying that data follow a Gaussian distribution, the mean antibody titre for each macaque species or national park was assessed for significance using one‐way ANOVA and post hoc Bonferroni test with a 95% confidence interval (CI) for the differences. A margin (mean antibody titre ±0.1) for each mosquito species was determined for the categorisation of individuals into low (mean − 0.1) and high (mean + 0.1) exposed groups as described by Aka et al. (2020). Pearson correlation coefficient of exposure was compared using the Kruskal–Wallis test for group differences and the post hoc Mann–Whitney test (95% CI) for the pairwise comparison. A previous study by Tongthainan et al. (2020) on the archived macaque sera found DENV and ZIKV neutralising antibody titres primarily on *M. arctoides* of KK. Thus, we compared (independent *t*‐test at 95% CI) anti‐SGE IgG titres between arbovirus DENV1‐4 and ZIKV‐positive and ZIKV‐negative *M. arctoides* groups in KK. Pearson correlation coefficients were determined to measure cross‐reactivity between IgG antibodies or antigenic variations between mosquito species under study. The statistical tests were carried out using GraphPad Prism version 8.4.3 (GraphPad Software, La Jolla, California, USA, www.graphpad.com) and SPSS® version 23.0.0.0 (IBM, Chicago, USA) software. For all statistical analyses, significance was considered if *p* ≤ 0.05.

## RESULTS

### Mosquito salivary gland proteins

Bradford assay of the SGE antigen showed the following protein concentrations in one batch of pooled SGE (100 pairs): 4.33 mg/mL for *Cx. quinquefasciatus*, 2.48 mg/mL for *Ae. albopictus* and 2.14 mg/mL for *Ae. aegypti*.

### Macaque anti‐SGE antibody titres

**FIGURE 2:**
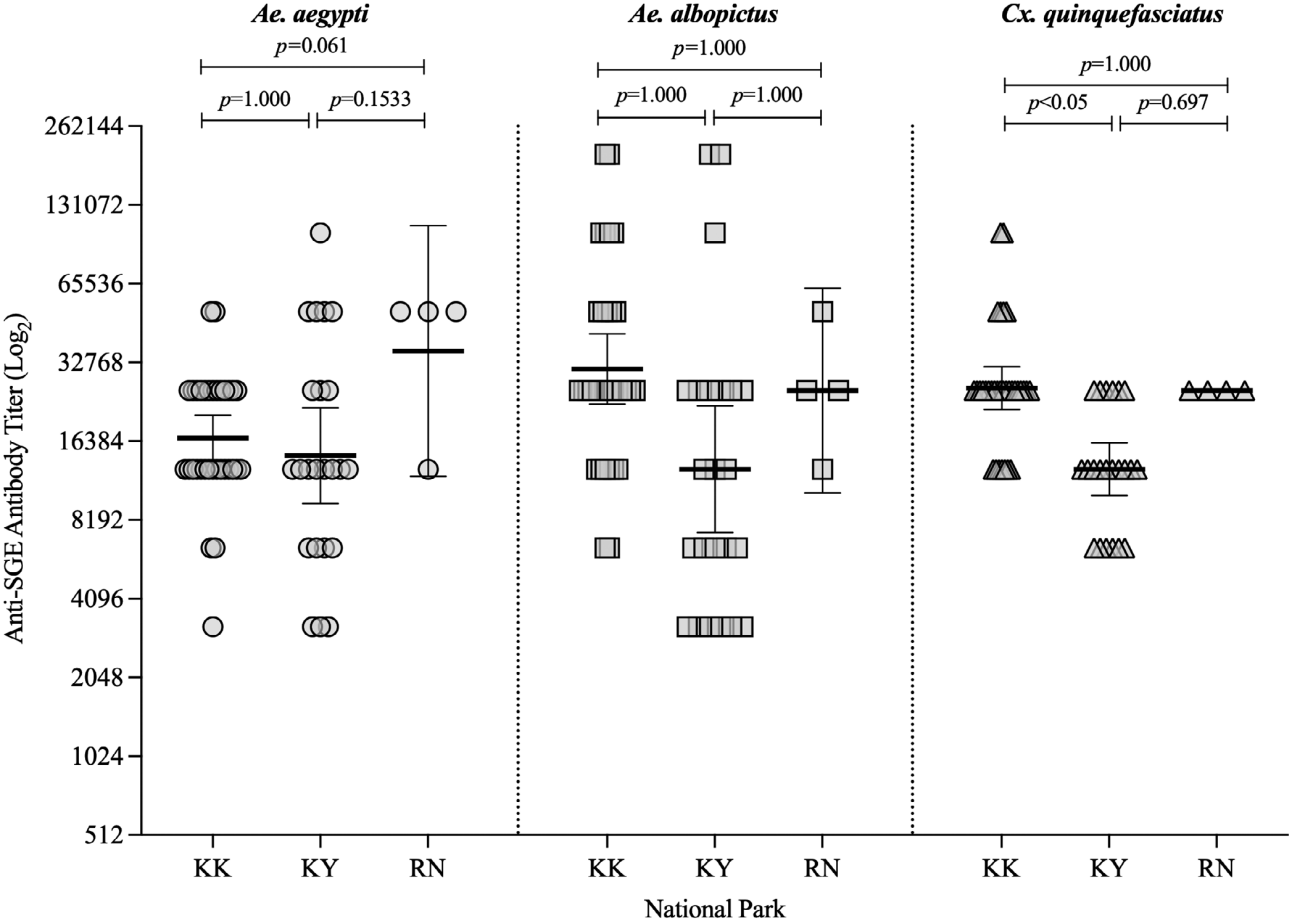
Immunoglobulin G SGE (salivary gland extract) antibody endpoint titres (log_2_) to three mosquito species: *Aedes aegypti* (circle), *Ae. albopictus* (square) and *Culex quinquefasciatus* (triangle) from national park‐dwelling macaques determined by ELISA. Serum samples were collected from *M. leonina*, *M. arctoides* and *M. fascicularis* from Khao Yai (KY) National Park in Nakhon Ratchasima, Kaeng Krachan (KK) National Park in Prachuap Khiri Khan and Mu Ko Ranong (RN) National Park in Ranong, respectively. Horizontal bars show the geometric mean with 95% CI. Differences between macaque species/national parks were tested using one‐way ANOVA and post hoc Bonferroni test (*p* values are indicated in the top panel).

The anti‐SGE antibody profiles of *M. arctoides*, *M. leonina* and *M. fascicularis* to specific mosquito antigens in the collection sites were examined using an endpoint titre ELISA‐based assay (Figure 2). Here, antibodies to *Ae. aegypti*, *Ae. albopictus* and *Cx. quinquefasciatus* were present in 60 macaques from KK, KY and RN National Parks. The mean anti‐*Cx. quinquefasciatus* antibody titre significantly differed between national parks (or macaque species) (*p* = 0.003). Specifically, the mean antibody titre to *Cx. quinquefasciatus* was higher in *M. arctoides* from KK than in *M. leonina* from KY (one‐way ANOVA, post hoc Bonferroni test, *p* = 0.002, Figure 2). No significant differences were noted between sites/macaque species for immune responses to *Ae. aegypti* (*p* = 0.066) and *Ae. albopictus* (*p* = 0.584). Antibody titres (log_2_) to *Ae. aegypti* SGE was highest in *M. fascicularis* from RN (geometric mean titre [GMT, (log_2_)] 36,204, 95% CI 12,016–109,084), followed by KK (GMT 19,491, 95% CI 13,726–20,609) and KY (GMT 14,440, 95% CI 9483–21,989) (Figure 2, panel 1; Supplementary Information S1, Tables S1 and S2). For anti‐*Ae. albopictus* SGE titres (log_2_), the highest to lowest titres were observed in KK (GMT 30,927, 95% CI 22,708–42,120), RN (GMT 25,600, 95% CI 10,402–63,001) and KY (GMT 12,800, 95% CI 7333–22,344) (Figure 2, panel 2; Supplementary Information S1, Tables S1 and S2). Lastly, antibody titres (log_2_) to *Cx. quinquefasciatus* SGE were observed in KK (GMT 26,143, 95% CI 21,636–31,590), RN (GMT 25,600, 95% CI 25,600–25,600) and KY (GMT 12,800, 95% CI 10,147–16,147) (Figure 2, panel 3; Supplementary Information S1, Tables S1 and S2).

### Mosquito bite exposure prevalence

The macaques were classified based on the immune response to the SGE antigen to characterise exposure to mosquito bites. The mean titre (log_2_) ± 0.1 was the threshold value to categorise individuals into low or high mosquito bite exposure groups (Aka et al., 2020). The seroprevalence of differential exposure to the feeding of mosquito species was presented as a proportion of SGE antibody‐positive macaque species. Seroprevalence of high exposure to *Cx. quinquefasciatus* between macaque species/national parks was significantly different (*p* < 0.05), specifically between KK (79%, 26 of 33) and KY (30%, 7 of 23) (*p* < 0.05) and between KY (30%, 7 of 23) and RN (100%, 4 of 4) (*p* = 0.027). KK‐inhabiting macaques were highly exposed primarily to *Cx. quinquefasciatus* (79%, 26 of 33), compared to the bites of *Ae. aegypti* (45%, 15 of 33) and *Ae. albopictus* (33%, 11 of 33). Higher exposure against *Cx. quinquefasciatus* (100%, 4 of 4) was also detected in *M. fascicularis*, followed by *Ae. aegypti* (75%, 3 of 4) and *Ae. albopictus* (25%, 1 of 4). For *M. leonina*, high exposure rates to *Ae. aegypti* (35%, 8 of 23) were observed, followed by its contact with *Cx. quinquefasciatus* (30%, 7 of 23) and *Ae. albopictus* (13%, 3 of 23); however, they were not significantly different (*p* > 0.05).

### *Arbovirus neutralisation titres and exposure to bites of* Aedes *vectors*

**FIGURE 3:**
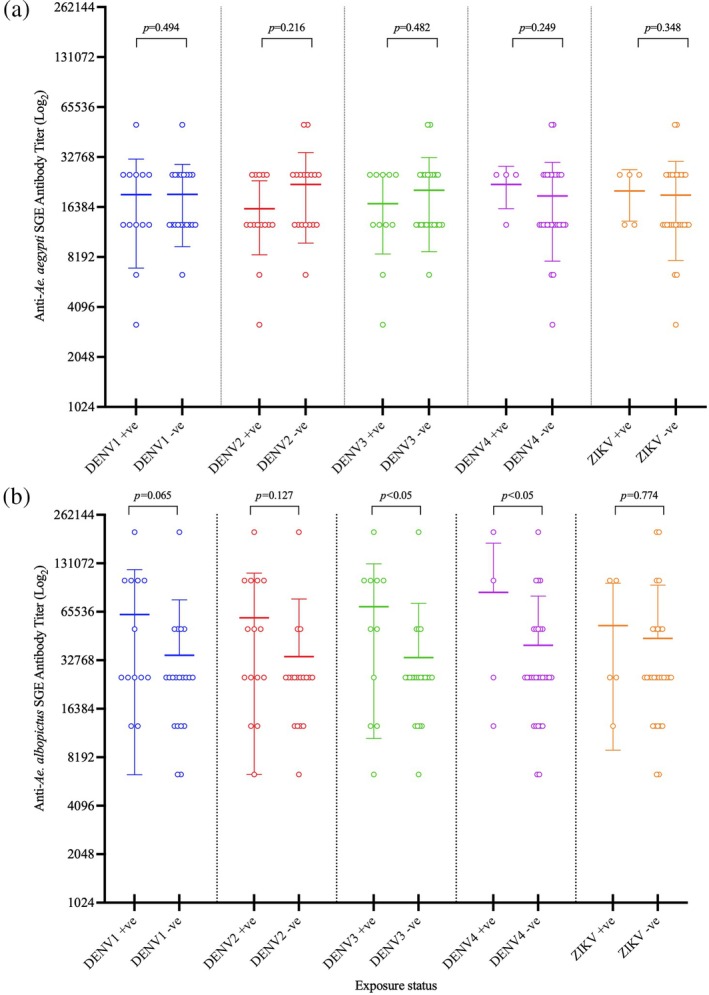
Comparison of salivary gland extract antibody titres (log_2_) against *Aedes aegypti* (a) and *Ae. albopictus* (b) between dengue virus/DENV—(a) and Zika virus/ZIKV—(b) positive (+ve) and ‐negative (−ve) stump‐tailed macaques (*Macaca arctoides*) from Kaeng Krachan National Park (*p* values are indicated in the top panel).

When comparing IgG levels between DENV‐ and ZIKV‐exposed and non‐exposed groups in the anti‐*Ae. aegypti* (Figure 3a) and *Ae. albopictus* (Figure 3b) groups, we detected significantly higher immune responses against *Ae. albopictus* in *M. arctoides* positive with DENV3 (independent *t* test, *p* = 0.034) and DENV4 (independent *t* test, *p* = 0.026) than the arbovirus‐negative macaque group. The association of DENV3 neutralisation titres against macaques' anti‐SGE antibody titres to arbovirus vector, *Ae. albopictus* revealed a significant correlation (*r* = 0.404, *p* = 0.022) (Table 1).

**TABLE 1:** Comparison of IgG antibody response against *Aedes* salivary gland extracts in *Macaca arctoides* serum samples with dengue virus/DENV1‐4 and Zika virus/ZIKV neutralisation titres from KK National Park.

	DENV1	DENV2	DENV3	DENV4	ZIKV
*Ae. aegypti*	0.033 (0.853)	−0.060 (0.739)	0.029 (0.875)	0.119 (0.508)	0.141 (0.442)
*Ae. albopictus*	0.300 (0.090)	0.247 (0.165)	0.404 (0.022)*	0.193 (0.282)	0.130 (0.477)

*Note*: The upper values are Pearson correlation values, and the values below, enclosed in parentheses, are the *p* values (*p value ≤ 0.05).

### Cross‐reaction of antibodies between mosquito species

**FIGURE 4:**
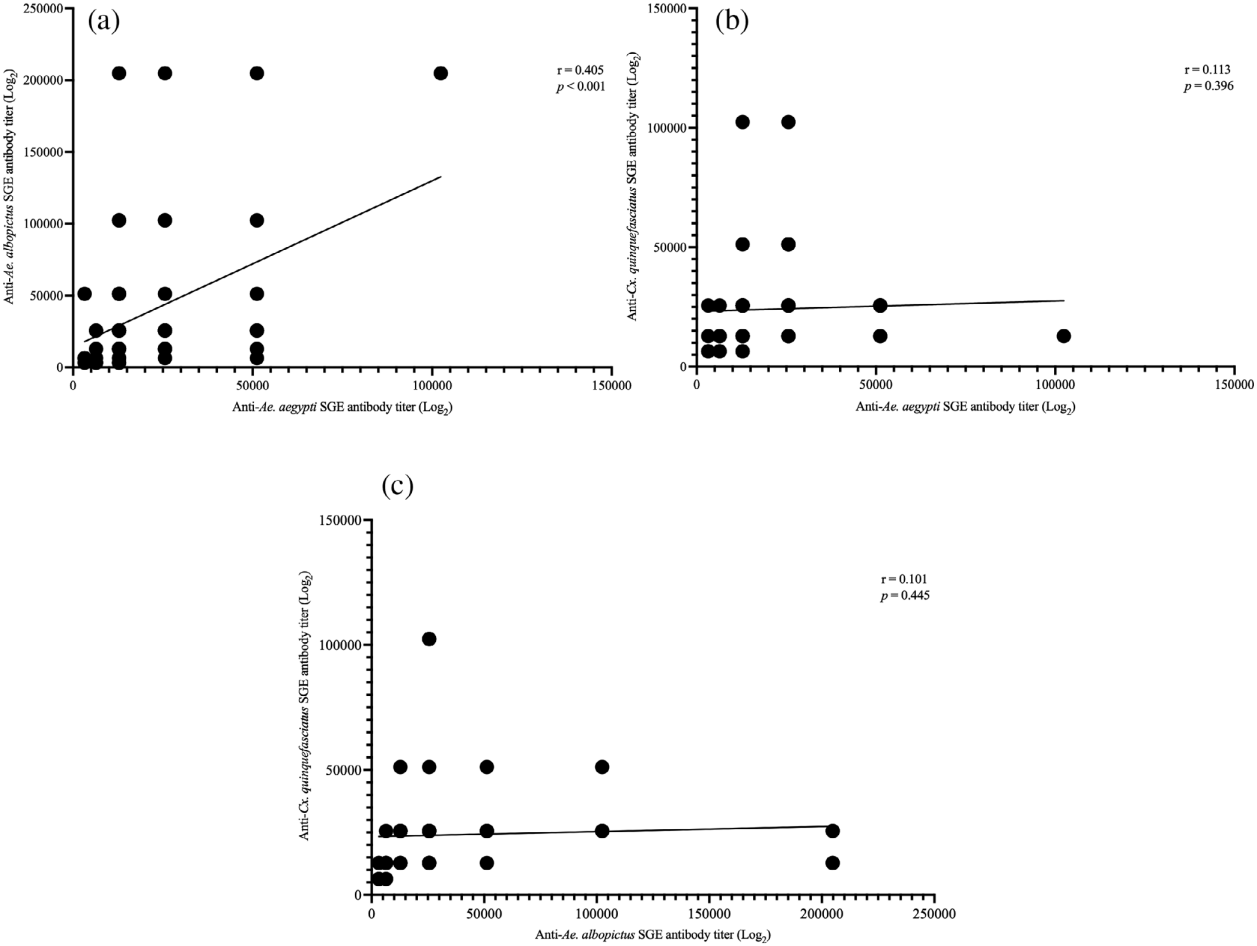
Pairwise correlation (Pearson coefficient *r*, *p* values) among IgG‐SGE (salivary gland extract) antibody titres (log_2_) against mosquito species in national park‐dwelling macaques in Thailand: (a) *Aedes aegypti* and *Ae. albopictus*, (b) *Ae. aegypti* and *Culex quinquefasciatus* and (c) *Ae. albopictus* and *Cx. quinquefasciatus*.

The IgG antibody levels against the three salivary antigens were compared for each macaque individually in the three national parks using a Pearson correlation test, and the corresponding *p* values were determined. Moderate cross‐reactivity of macaque IgG antibody responses was observed between *Ae. aegypti* and *Ae. albopictus* (*r* = 0.405, *p* = 0.001) (Figure 4). No significant cross‐reactivity was observed between the mosquito genera *Aedes* and *Culex* (Figure 4b,c).

## DISCUSSION

Here, we present a sero‐immunological analysis of the macaque antibody response to the salivary antigen of *Ae. aegypti, Ae. albopictus* and *Cx. quinquefasciatus* using ELISA. The results have practical public health relevance for *Aedes*‐borne diseases, which are common in most tropical and subtropical countries. Specifically, our results detected reactivity to crude SGE proteins, although there is cross‐reactivity between *Aedes* mosquitoes, suggesting that the IgG response to vector saliva may represent a proxy exposure of vertebrate animals to mosquito bites.

Previously, NHPs (*Erythrocebus patas* [Schreber, 1774] [Primates: Cercopithecidae] and *M. mulatta* [Zimmermann, 1780] [Primates: Cercopithecidae]) in Puerto Rico were found seropositive to SGEs of *Ae. aegypti* and *Cx. quinquefasciatus* in addition to *Ae. mediovittatus* (Bigot, 1861) (Diptera: Culicidae) and *Ae. tritaeniorhynchus* (Wiedemann, 1821) (Diptera: Culicidae) (Hemme et al., 2016). Our findings corroborate those of Hemme et al. (2016), who found that *Ae. aegypti* and *Cx. quinquefasciatus* fed upon macaques, thus highlighting the role of NHPs and their susceptibility to dengue infection (Gwee et al., 2021) and transmission of mosquito‐borne arboviral diseases. Other mosquito species, such as *Anopheles* spp. malaria vectors (*An. minimus* [Theobald, 1901] [Diptera: Culicidae] and *An. dirus* [Peyton & Harrison, 1979] [Diptera: Culicidae]) might also have fed upon the macaques as Fungfuang et al. 2020 found *Plasmodium*‐infected NHPs; thus, potentially explaining the higher titres observed in our study. Also, *An. latens* [Sallum et al., 2005] [Diptera: Culicidae] and *An. introlatus* [Colless, 1957] [Diptera: Culicidae] are proven vectors of monkey malaria in southern Thailand (Yanmanee et al., 2023). In addition, other biting dipterans could be present in national parks, such as Stomoxyini flies (Changbunjong et al., 2012). NHPs seropositive to dengue and Zika have been found in Thailand, suggesting sylvatic cycles might also occur there (Nakgoi et al., 2014), although there have been no sylvatic mosquito or viral characterisation studies (Valentine et al., 2019).

Given that concurrent mosquito abundance and landing rate on NHPs in our study sites were not conducted, we reviewed vector bionomics and its associations with ecology, environment, land development and human incursions. The mosquito species selected are locally incriminated as vectors of mosquito‐borne diseases and are widely distributed across regions in Thailand (Saeung, 2012). Predominant mosquito genera collected in Thailand forest parks were mainly *Culex*, *Aedes*, *Anopheles* and *Uranotenia* (Srisuka et al., 2022; Thongsripong et al., 2013). Discarded containers are considered key mosquito development sites, increasing the risk of vector abundance (Sahavechaphan et al., 2020; Thammapalo et al., 2021). Trash (e.g. bottles and chip bags) left by visitors of KY National Park prompted Thailand's Minister of Natural Resources and Environment to implement stricter measures on waste disposal (Compton, 2020), as these items provide ideal mosquito development habitats and discarded food attract NHPs. In Brazil, there were reports of the coexistence of urban and sylvatic mosquitoes in forested areas (Ahebwa et al., 2023; Hendy et al., 2020; Rosa‐Silva et al., 2023). In the KK National Park, human settlements may also contribute to the presence of peridomestic vectors in the fragmented forest areas (Thongsripong et al., 2013) and could account for the observed anti‐*Ae. aegypti* antibody titres. *Culex quinquefasciatus* and *Ae. albopictus* also were collected abundantly in a breeding facility of long‐tailed macaques (Novianto et al., 2022). Higher IgG antibody levels were observed in Côte d'Ivoire, particularly in areas with intensive agricultural activities associated with mosquito development sites (collection buckets) for *Ae. albopictus* in the rubber plantation (Yobo et al., 2018). It is hypothesised that mosquito vectors access development sites in the national parks and will feed upon available humans or NHPs for egg development and continuation of the life cycle. In terms of feeding behaviour, mosquito body size affects its bloodmeal, wherein smaller *Ae. aegypti* and *Ae. albopictus* mosquitoes have adapted to more frequent host‐seeking behaviour and multiple blood meals (Farjana & Tuno, 2013). *Aedes albopictus*, in particular, exhibits exophagic and opportunistic feeding with high host plasticity (Delatte et al., 2010). *Culex quinquefasciatus* exhibits a longer probing time on mammalian hosts than on birds (Ribeiro, 2000). This behaviour of the *Aedes* and *Culex* mosquitoes could trigger a more intense immune response since more saliva proteins are injected into the host. In rural Thai villages, *Ae. aegypti* preferentially fed on humans with a rare occurrence of bloodmeals obtained from other animal hosts, such as bovine, cat, chicken, rat and swine (Ponlawat & Harrington, 2005). The occurrence of mixed bloodmeal reflects multiple feeding patterns based on the available host species in the area (Pengsakul et al., 2017). Anthropophilic *Ae. aegypti* and *Cx. quinquefasciatus* from villages in Koh Chang Island, Thailand, were detected with multiple host blood‐feeding patterns, including monkeys (Khaklang & Kittayapong, 2014). *Aedes albopictus*, on the other hand, exhibits greater zoophagy than anthropophagy, contributing to its capacity as a bridge vector of mosquito‐borne arboviruses (Fikrig et al., 2023; Pereira Dos Santos et al., 2018; Richards et al., 2006). Aside from vector biology, host activity (such as movement, resting behaviour and attractiveness) may also contribute to variations in the frequency of mosquito bite exposure. The ecological shift of stump‐tailed and long‐tailed macaques from the mountains to the human community was observed at Wat Tham Khao Daeng, Amphoe Ron Phibun, Nakhonsithammarat Province (Malaivijitnond & Hamada, 2005). Stump‐tailed macaques were more adapted to humans and stayed longer in the village to play, groom and mount on partners than long‐tailed macaques (Malaivijitnond & Hamada, 2005). The 16 species of macaques found primarily in southern Asia (Napier & Napier, 1985) are omnivorous, adaptable to almost any ecological niche and have unregulated, extensive and often close contact with humans, such as recreation travellers, tourists or religious worshippers. Such scenarios are commonly observed in temples and national parks in Southeast Asia (Jones‐Engel et al., 2006), as well as in Thailand (Malaivijitnond & Hamada, 2005).

A previous study by Tongthainan et al. (2020) on the archived sera used in the current study detected neutralising antibody titres to DENV and ZIKV, primarily in *M. arctoides* from KK National Park. As such, we compared the IgG levels against the SGE of virus infected and uninfected *Aedes* vectors in KK‐inhabiting macaques. We found limited associations and differences between the immune reaction to mosquito SGE and neutralisation titres to Zika and dengue serotypes. Salivary proteins work as an immunosuppressant at high concentrations but modulate the immune response at low concentrations, specifically down‐regulating TH1 (T helper 1) and antiviral cytokines and unaffected or amplified TH2 (T helper 2) cytokines (Schneider & Higgs, 2008). Previous studies found that a high immune response against mosquito saliva was associated with dengue‐infected individuals, concluding the accuracy of IgG antibodies for the risk of dengue infection (Cardenas et al., 2019; Londono‐Renteria et al., 2013). Machain‐Williams et al. (2012) also observed that DENV2‐infected children have higher antibody titres to *Ae. aegypti* secreted saliva. Consequently, the observed different findings may reside in the antigen utilised to capture the antibodies. In contrast to extracted saliva from the earlier study, we used crude SGE proteins in the baseline detection of IgG antibodies; SGE contains extraneous proteins not secreted in the saliva (Wongkamchai et al., 2010). These protein families are specific or ubiquitously shared at different taxonomic levels; families, genera and species of mosquitoes and other biting dipterans (sand fly, black fly, midge, stable fly, etc.), as well as other hematophagous arthropods like ticks (Ribeiro et al., 2010; Sagna et al., 2018). The shared proteins in the crude SGE could support the cross‐reactivity of immune responses between mosquito species, as seen in several studies between *Ae. aegypti*, *Ae. albopictus*, *Cx. quinquefasciatus* and even with *An. minimus* due to shared salivary antigens (Doucoure et al., 2012; Doucoure et al., 2013; Fontaine et al., 2011; Wongkamchai et al., 2010). In the current study, species‐relatedness could further corroborate the cross‐reactivity between the two *Aedes* spp. belonging to the same subgenus, *Stegomyia*. As in the earlier study of Hemme et al. (2016), they detected no significant mosquito‐specific cross‐reactivity between *Aedes* of different subgenera: *Ae. aegypti* (subgenus *Stegomyia*), *Ae. mediovittatus* (subgenus *Gymnometopa*) and *Ae. taeniorhynchus* (subgenus *Ochlerotatus*) (Hemme et al., 2016).

There are several limitations in the study. In our study, macaque species was confounded by national park as only one species was studied from each park. Previous studies that sampled macaques in Thailand national parks (Aggimarangsee, 1992; Albert et al., 2013; Malaivijitnond & Yuzuru, 2008; Treesucon, 1988) found species‐specific localisation patterns with typically one macaque species being dominant in each park. This may be influenced by the varying environmental and ecological factors supporting species survival, as Lekagul & McNeely (1988) reported. In Thailand, long‐tailed macaques are the most commonly observed primate species among the 13 species present (Lekagul & McNeely, 1988). This species is especially prevalent in Southern Thailand, as reflected in our study (Aggimarangsee, 1992; Malaivijitnond & Yuzuru, 2008). Meanwhile, *M. leonina* and *M. arctoides* have been reported locally in Thailand (Albert et al., 2013; Malaivijitnond & Yuzuru, 2008; Treesucon, 1988). The current study has a low sample size, particularly for *M. fascicularis* at RN National Park (*n* = 4), which may limit the generalizability of the findings. A larger sample size is recommended for a more comprehensive and conclusive result to strengthen the understanding of macaque distribution patterns in national parks.

Our study focused on macaque seroprevalence to Culicine SGE, and its evaluation could be improved by addressing the cross‐reactions observed. The way forward may include ELISA experiments with species‐specific proteins or peptides. For further study, the Nterm‐34 kDA peptide for *Ae. aegypti* may provide a more specific antibody response for *Ae. aegypti* exposure (Fustec et al., 2021). The duration to which the crude SGE may be used to measure mosquito exposure in seroepidemiological studies or evaluate the effectiveness of vector control interventions is unclear. Some studies have found that the anti‐gSG6 antibody response may serve to assess the impact of interventions in the short term (less than a year) (Drame et al., 2010). In contrast, other studies have found that the antibody response can assess the impact of interventions in the longer term (Idris et al., 2017). The need to develop an alternative biomarker for vector control applications to gSG6 is highlighted in these studies. Entomological data on mosquito population, density and natural infectivity in national parks are needed to support and confirm macaque exposure to Culicine mosquitoes.

Like human hosts, macaques are exposed to mosquito blood‐feeding in the wild and may develop an immune response against immunogenic salivary proteins. In our study, we detected immune responses against mosquito bites in macaques from national parks in Thailand. This aids in establishing the utility of salivary biomarkers for NHPs as a proxy for mosquito‐monkey contact. Serological assays provide an alternative and simple method to assess the human or monkey‐biting rates due to ethics regulations of animal experimentation and limitations of animal‐baited traps, such as low trap efficiency, logistical challenges and, more importantly, concerns for animal welfare. Serological data could serve as a basis for risk management in national parks and tourist sites where people can become infected when encroaching on these habitats and are bitten by mosquitoes carrying arboviruses or if infected forest mosquitoes move into areas of human habitation to obtain a blood meal. This could aid public health authorities in mitigating or curbing the sylvatic transmission cycle and spillover into an urban transmission cycle through mosquito management techniques such as biocontrol using various natural predators, environmental management or modification, landscape and urban planning, coordination and cooperation between policymakers, law enforcement officers and pubic members, public education and awareness campaigns, relocation of farmlands further away from the forests, or avoidance of outdoor activities during the feeding period of vectors (Lee et al., 2022).

## CONCLUSION

Our findings provide baseline information about the mosquito bite exposure of macaques found in Thailand's national parks despite detecting cross‐reactive immune responses. The study presents the seroprevalence of culicine mosquito saliva for macaques, complementing currently available entomological techniques in determining vector‐host interactions.

It suggests a risk of sylvatic transmission of known or novel pathogens between animal hosts and humans living within or near the national parks or those merely visiting the forested tourist areas. Through studying anti‐SGE immune responses, areas with heightened host‐vector contact can be identified or mapped to enhance surveillance and raise vigilance to prevent outbreak occurrence. Nevertheless, data gathered could serve as a basis for risk management in national parks by mitigating the risk of vector‐host interfaces, such as recommending tourists use repellents or bed nets for overnight visitors or to take prophylaxis.

## AUTHOR CONTRIBUTIONS

**Ariza Minelle A. Aguila:** Investigation; methodology; formal analysis; writing – original draft; funding acquisition. **Kobporn Boonnak:** Conceptualization; supervision; writing – review and editing; funding acquisition. **Daraka Tongthainan:** Writing – review and editing; resources. **Onrapak Reamtong:** Investigation; writing – review and editing; formal analysis; writing – original draft. **Sarocha Suthisawat:** Writing – review and editing; investigation. **Oranit Likhit:** Investigation; writing – review and editing. **Wirasak Fungfuang:** Resources; writing – review and editing. **Jeffrey Hii:** Investigation; writing – original draft; formal analysis; writing – review and editing. **Patchara Sriwichai:** Conceptualization; supervision; writing – review and editing; formal analysis.

## FUNDING INFORMATION

The research was funded by the National Science and Technology Development Agency (NSTDA), Thailand, through the Asia joint research program (grant number FDA‐CO‐2562‐9880‐TH) to KB and fully supported by Deutscher Akademischer Austauschdienst (DAAD) German Academic Exchange Service to AA.

## CONFLICT OF INTEREST STATEMENT

The authors declare no conflict of interest. The funders had no role in the study's design, in the collection, analyses or interpretation of data, in the writing of the manuscript, or in the decision to publish the results.

## ETHICS APPROVAL AND CONSENT TO PARTICIPATE

The Institutional Animal Care and Use Committee of Mahidol University in Thailand granted ethical approval for this study (FTM‐ACUC 003/2019E).

## CONSENT FOR PUBLICATION

Not applicable.

## Supporting information


Supplementary Information S1.
**Table S1**. Mean anti‐mosquito (*Aedes aegypti*, *Ae. albopictus* and *Culex quinquefasciatus*) SGE (salivary gland extract) antibody endpoint titres (Log_2_) of *Macaca arctoides*, *M. leonina* and *M. fascicularis* in Thailand.
**Table S2**. Geometric mean anti‐mosquito (*Aedes aegypti*, *Ae. albopictus* and *Culex quinquefasciatus*) SGE (salivary gland extract) antibody endpoint titres (Log_2_) of *Macaca arctoides*, *M. leonina* and *M. fascicularis* in Thailand.

## Data Availability

Raw data are available from the corresponding author upon request. Data sharing in DOI: http://datadryad.org/stash/share/Px5zSbNemOIUFEX6SClWb09Nijgn0LLmE2pTB3XXYQk.

## References

[mve12779-bib-0001] Aggimarangsee, N. (1992) Survey for semi‐tame colonies of macaques in Thailand (doctoral dissertation, Mahidol University). Natural History Bulletin of the Siam Society, 40(2), 103–166.

[mve12779-bib-0002] Ahebwa, A. , Hii, J. , Neoh, K.B. & Chareonviriyaphap, T. (2023) *Aedes aegypti* and *Aedes albopictus* (Diptera: Culicidae) ecology, biology, behaviour, and implications on arbovirus transmission in Thailand: review. One Health, 16, 100555. Available from: 10.1016/j.onehlt.2023.100555 37363263 PMC10288100

[mve12779-bib-0003] Aka, K.G. , Traore, D.F. , Sagna, A.B. , Zoh, D.D. , Assi, S.B. , Tchiekoi, B.N. et al. (2020) Pattern of antibody responses to *Plasmodium falciparum* antigens in individuals differentially exposed to *Anopheles* bites. Malaria Journal, 19(1), 83. Available from: 10.1186/s12936-020-03160-5 32085710 PMC7033907

[mve12779-bib-0004] Albert, A. , Hambuckers, A. , Culot, L. , Savini, T. & Huynen, M.‐C. (2013) Frugivory and seed dispersal by Northern Pigtailed Macaques (*Macaca leonina*), in Thailand. International Journal of Primatology, 34(1), 170–193. Available from: 10.1007/s10764-012-9649-5

[mve12779-bib-0005] Boulanger, D. , Doucoure, S. , Grout, L. , Ngom, A. , Rogerie, F. , Cornelie, S. et al. (2011) Immunoglobulin G antibody profiles against *Anopheles* salivary proteins in domestic animals in Senegal. Journal of Medical Entomology, 48(3), 691–693. Available from: 10.1603/me10183 21661332

[mve12779-bib-0006] Cardenas, J.C. , Drame, P.M. , Luque‐Burgos, K.A. , Berrio, J.D. , Entrena‐Mutis, E. , Gonzalez, M.U. et al. (2019) IgG1 and IgG4 antibodies against *Aedes aegypti* salivary proteins and risk for dengue infections. PLoS One, 14(1), e0208455. Available from: 10.1371/journal.pone.0208455 30601814 PMC6314615

[mve12779-bib-0007] Changbunjong, T. , Weluwanarak, T. , Ratanakorn, P. , Sungvornyothin, S. , Sriwichai, P. , Sumruayphol, S. et al. (2012) Distribution and abundance of Stomyxyini flies (Diptera: Muscidae) in Thailand. The Southeast Asian Journal of Tropical Medicine and Public Health, 43(6), 1400–1410. 23413703

[mve12779-bib-0008] Coleman, J. , Juhn, J. & James, A.A. (2007) Dissection of midgut and salivary glands from *Ae. aegypti* mosquitoes. Journal of Vision, 5, 228. Available from: 10.3791/228 PMC2557086 18979026

[mve12779-bib-0009] Compton, N. (2020) This Thai national park was tired of visitors leaving trash, so the government mailed it back to them. The Washington Post. Retrieved October 1 from https://www.washingtonpost.com/travel/2020/09/18/tourist-trash-mail/.

[mve12779-bib-0010] Delatte, H. , Desvars, A. , Bouetard, A. , Bord, S. , Gimonneau, G. , Vourc'h, G. et al. (2010) Blood‐feeding behavior of Aedes albopictus, a vector of Chikungunya on La Reunion. Vector‐Borne and Zoonotic Diseases, 10(3), 249–258. Available from: 10.1089/vbz.2009.0026 19589060

[mve12779-bib-0011] Doucoure, S. , Cornelie, S. , Patramool, S. , Mouchet, F. , Demettre, E. , Seveno, M. et al. (2013) First screening of *Aedes albopictus* immunogenic salivary proteins. Insect Molecular Biology, 22(4), 411–423. Available from: 10.1111/imb.12032 23714164

[mve12779-bib-0012] Doucoure, S. , Mouchet, F. , Cornelie, S. , DeHecq, J.S. , Rutee, A.H. , Roca, Y. et al. (2012) Evaluation of the human IgG antibody response to *Aedes albopictus* saliva as a new specific biomarker of exposure to vector bites. PLoS Neglected Tropical Diseases, 6(2), e1487. Available from: 10.1371/journal.pntd.0001487 22363823 PMC3283547

[mve12779-bib-0013] Drame, P.M. , Poinsignon, A. , Besnard, P. , Le Mire, J. , Dos‐Santos, M.A. , Sow, C.S. et al. (2010) Human antibody response to *Anopheles gambiae* saliva: an immuno‐epidemiological biomarker to evaluate the efficacy of insecticide‐treated nets in malaria vector control. The American Journal of Tropical Medicine and Hygiene, 83(1), 115–121. Available from: 10.4269/ajtmh.2010.09-0684 20595489 PMC2912587

[mve12779-bib-0014] Farjana, T. & Tuno, N. (2013) Multiple blood feeding and host‐seeking behavior in *Aedes aegypti* and *Aedes albopictus* (Diptera: Culicidae). Journal of Medical Entomology, 50(4), 838–846. Available from: 10.1603/me12146 23926783

[mve12779-bib-0015] Fikrig, K. , Rose, N. , Burkett‐Cadena, N. , Kamgang, B. , Leisnham, P.T. , Mangan, J. et al. (2023) *Aedes albopictus* host odor preference does not drive observed variation in feeding patterns across field populations. Scientific Reports, 13(1), 130. Available from: 10.1038/s41598-022-26591-3 36599854 PMC9813369

[mve12779-bib-0016] Fontaine, A. , Pascual, A. , Orlandi‐Pradines, E. , Diouf, I. , Remoue, F. , Pages, F. et al. (2011) Relationship between exposure to vector bites and antibody responses to mosquito salivary gland extracts. PLoS One, 6(12), e29107. Available from: 10.1371/journal.pone.0029107 22195000 PMC3237593

[mve12779-bib-0077] Fungfuang, W. , Udom, C. , Tongthainan, D. , Kadir, K.A. , & Singh, B. (2020) Malaria parasites in macaques in Thailand: stump‐tailed macaques (Macaca arctoides) are new natural hosts for *Plasmodium knowlesi*, *Plasmodium inui*, *Plasmodium coatneyi* and *Plasmodium fieldi* . Malaria Journal, 19(1). Available from: 10.1186/s12936-020-03424-0 PMC7528273 33004070

[mve12779-bib-0017] Fustec, B. , Phanitchat, T. , Aromseree, S. , Pientong, C. , Thaewnongiew, K. , Ekalaksananan, T. et al. (2021) Serological biomarker for assessing human exposure to *Aedes* mosquito bites during a randomized vector control intervention trial in northeastern Thailand. PLoS Neglected Tropical Diseases, 15(5), e0009440. Available from: 10.1371/journal.pntd.0009440 34043621 PMC8189451

[mve12779-bib-0080] Gutiérrez‐López, R. , Logan, J.G. , & Martínez‐de la Puente, J. (Eds.). (2022) Ecology of diseases transmitted by mosquitoes to wildlife. Wageningen Academic Publishers, Netherlands. Available from: 10.3920/978-90-8686-931-2

[mve12779-bib-0019] Gwee, S.X.W. , St John, A.L. , Gray, G.C. & Pang, J. (2021) Animals as potential reservoirs for dengue transmission: a systematic review. One Health, 12, 100216. Available from: 10.1016/j.onehlt.2021.100216 33598525 PMC7868715

[mve12779-bib-0020] Han, B.A. , Majumdar, S. , Calmon, F.P. , Glicksberg, B.S. , Horesh, r. , Kumar, A. et al. (2019) Confronting data sparsity to identify potential sources of Zika virus spillover infection among primates. Epidemics, 27, 59–65. Available from: 10.1016/j.epidem.2019.01.005 30902616

[mve12779-bib-0021] Hawkes, F. , Manin, B.O. , Ng, S.H. , Torr, S.J. , Drakeley, C. , Chua, T.H. et al. (2017) Evaluation of electric nets as means to sample mosquito vectors host‐seeking on humans and primates. Parasites & Vectors, 10(1), 338. Available from: 10.1186/s13071-017-2277-3 28720113 PMC5516363

[mve12779-bib-0022] Hemme, R.R. , Poole‐Smith, B.K. , Hunsperger, E.A. , Felix, G.E. , Horiuchi, K. , Biggerstaff, B.J. et al. (2016) Non‐human primate antibody response to mosquito salivary proteins: implications for dengue virus transmission in Puerto Rico. Acta Tropica, 164, 369–374. Available from: 10.1016/j.actatropica.2016.08.027 27593498

[mve12779-bib-0023] Hendy, A. , Hernandez‐Acosta, E. , Chaves, B.A. , Fe, N.F. , Valerio, D. , Mendonca, C. et al. (2020) Into the woods: changes in mosquito community composition and presence of key vectors at increasing distances from the urban edge in urban forest parks in Manaus, Brazil. Acta Tropica, 206, 105441. Available from: 10.1016/j.actatropica.2020.105441 32173316 PMC7184314

[mve12779-bib-0024] Idris, Z.M. , Chan, C.W. , Mohammed, M. , Kalkoa, M. , Taleo, G. , Junker, K. et al. (2017) Serological measures to assess the efficacy of malaria control programme on Ambae Island, Vanuatu. Parasites & Vectors, 10(1), 204. Available from: 10.1186/s13071-017-2139-z 28441959 PMC5405492

[mve12779-bib-0025] Jones‐Engel, L. , Engel, G. , Heidrich, J. , Chalise, M. , Poudel, N. , Viscidi, R. et al. (2006) Temple monkeys and health implications of commensalism, Kathmandu, Nepal. Emerging Infectious Diseases, 12(6), 900–906. 16707044 10.3201/eid1206.060030 PMC3373059

[mve12779-bib-0026] Khaklang, S. & Kittayapong, P. (2014) Species composition and blood meal analysis of mosquitoes collected from a tourist Island, Koh Chang, Thailand. Journal of Vector Ecology, 39, 448–452. 25424275 10.1111/jvec.12122

[mve12779-bib-0027] Kielkopf, C.L. , Bauer, W. & Urbatsch, I.L. (2020) Bradford assay for determining protein concentration. Cold Spring Harbor Protocols, 2020(4), 102269. Available from: 10.1101/pdb.prot102269 32238597

[mve12779-bib-0028] Lakhotia, D. , Tun, Y.M. , Mongkol, N. , Likhit, O. , Suthisawat, S. , Mangmee, S. et al. (2023) A Serosurvey of Japanese encephalitis virus in monkeys and humans living in proximity in Thailand. Viruses, 15(5), 1125. Available from: 10.3390/v15051125 37243211 PMC10221860

[mve12779-bib-0029] Lee, W.C. , Cheong, F.W. , Amir, A. , Lai, M.Y. , Tan, J.H. , Phang, W.K. et al. (2022) Plasmodium knowlesi: the game changer for malaria eradication. Malaria Journal, 21(1), 140. Available from: 10.1186/s12936-022-04131-8 35505339 PMC9066973

[mve12779-bib-0030] Leitner, W. , Costero‐Saint Denis, A. & Wali, T. (2017) The site of the bite: addressing knowledge gaps in vector transmission of diseases. In: Arthropod vector: controller of disease transmission, Vol. 1. Amsterdam, Netherlands: Elsevier Inc., pp. 1–10.

[mve12779-bib-0031] Lekagul, B. & McNeely, J.A. (1988) Mammals of Thailand. Bangkok, Thailand: Darnsutha Press, pp. 478.

[mve12779-bib-0032] Londono‐Renteria, B. , Cardenas, J.C. , Cardenas, L.D. , Christofferson, R.C. , Chisenhall, D.M. , Wesson, D.M. et al. (2013) Use of anti‐*Aedes aegypti* salivary extract antibody concentration to correlate risk of vector exposure and dengue transmission risk in Colombia. PLoS One, 8(12), e81211. Available from: 10.1371/journal.pone.0081211 24312537 PMC3846924

[mve12779-bib-0033] Londono‐Renteria, B. , Drame, P.M. , Montiel, J. , Vasquez, A.M. , Tobon‐Castano, A. , Taylor, M. et al. (2020) Identification and pilot evaluation of salivary peptides from *Anopheles albimanus* as biomarkers for bite exposure and malaria infection in Colombia. International Journal of Molecular Sciences, 21(3), 691. Available from: 10.3390/ijms21030691 31973044 PMC7037407

[mve12779-bib-0034] Londono‐Renteria, B.L. , Eisele, T.P. , Keating, J. , James, M.A. & Wesson, D.M. (2010) Antibody response against *Anopheles albimanus* (Diptera: Culicidae) salivary protein as a measure of mosquito bite exposure in Haiti. Journal of Medical Entomology, 47(6), 1156–1163. Available from: 10.1603/me09240 21175067

[mve12779-bib-0035] Machain‐Williams, C. , Mammen, M.P., Jr. , Zeidner, N.S. , Beaty, B.J. , Prenni, J.E. , Nisalak, A. et al. (2012) Association of human immune response to *Aedes aegypti* salivary proteins with dengue disease severity. Parasite Immunology, 34(1), 15–22. Available from: 10.1111/j.1365-3024.2011.01339.x 21995849 PMC3240707

[mve12779-bib-0036] Malaivijitnond, S. & Hamada, Y. (2005) A new record of stump‐tailed macaques in Thailand and the sympatry with long‐tailed macaques. The Natural History Journal of Chulalongkorn University, 5, 93–96.

[mve12779-bib-0037] Malaivijitnond, S. , Vazquez, Y. & Hamada, Y. (2011) Human impact on long‐tailed macaques in Thailand. In: Monkeys on the edge. Cambridge, England: Cambridge University Press, pp. 118–158. Available from: 10.1017/CBO9780511974434.007

[mve12779-bib-0038] Malaivijitnond, S. & Yuzuru, H. (2008) Current situation and status of long‐tailed macaques (*Macaca fascicularis*) in Thailand. The Natural History Journal of Chulalongkorn University, 8(2), 185–204.

[mve12779-bib-0039] Malijan, R.P.B. , Mechan, F. , Braganza, J.C., Jr. , Valle, K.M.R. , Salazar, F.V. , Torno, M.M. et al. (2021) The seasonal dynamics and biting behavior of potential *anopheles* vectors of *Plasmodium knowlesi* in Palawan, Philippines. Parasites & Vectors, 14(1), 357. Available from: 10.1186/s13071-021-04853-9 34233742 PMC8261946

[mve12779-bib-0040] Mathieu‐Daude, F. , Claverie, A. , Plichart, C. , Boulanger, D. , Mphande, F.A. & Bossin, H.C. (2018) Specific human antibody responses to *Aedes aegypti* and *Aedes polynesiensis* saliva: a new epidemiological tool to assess human exposure to disease vectors in the Pacific. PLoS Neglected Tropical Diseases, 12(7), e0006660. Available from: 10.1371/journal.pntd.0006660 30040826 PMC6075770

[mve12779-bib-0041] Miot, E.F. , Calvez, E. , Aubry, F. , Dabo, S. , Grandadam, M. , Marcombe, S. et al. (2020) Risk of arbovirus emergence via bridge vectors: case study of the sylvatic mosquito *Aedes malayensis* in the Nakai district, Laos. Scientific Reports, 10(1), 7750. Available from: 10.1038/s41598-020-64696-9 32385369 PMC7210265

[mve12779-bib-0042] Nakgoi, K. , Nitatpattana, N. , Wajjwalku, W. , Pongsopawijit, P. , Kaewchot, S. , Yoksan, S. et al. (2014) Dengue, Japanese encephalitis and chikungunya virus antibody prevalence among captive monkey (*Macaca nemestrina*) colonies of northern Thailand. American Journal of Primatology, 76(1), 97–102. Available from: 10.1002/ajp.22213 24105916

[mve12779-bib-0043] Napier, J. & Napier, P. (1985) The natural history of the primates. Cambridge, Massachusetts, United States: MIT Press.

[mve12779-bib-0044] Narat, V. , Alcayna‐Stevens, L. , Rupp, S. & Giles‐Vernick, T. (2017) Rethinking human‐nonhuman primate contact and pathogenic disease spillover. EcoHealth, 14(4), 840–850. Available from: 10.1007/s10393-017-1283-4 29150826

[mve12779-bib-0045] Novianto, D. , Hadi, U.K. , Soviana, S. , Supriyono, S. , Rosmanah, L. & Darusman, H.S. (2022) Diversity of mosquito species and potential arbovirus transmission in long‐tailed macaque (*Macaca fascicularis*) breeding facilities. Veterinary World, 15(8), 1961–1968. Available from: 10.14202/vetworld.2022.1961-1968 36313848 PMC9615496

[mve12779-bib-0046] Pengsakul, T. , Sudsom, N. , Foakes, G. , Bhatt, K. , Eisenberg, M. & Siriyasatien, P. (2017) Molecular DNA identification of blood sources fed on for Culicine mosquitoes (Diptera: Culicidae) collected in the Songkhla province, southern Thailand. Songklanakarin Journal of Science and Technology, 39, 731–737.

[mve12779-bib-0047] Pereira Dos Santos, T. , Roiz, D. , Santos de Abreu, F.V. , Luz, S.L.B. , Santalucia, M. , Jiolle, D. et al. (2018) Potential of *Aedes albopictus* as a bridge vector for enzootic pathogens at the urban‐forest interface in Brazil. Emerg Microbes Infect, 7(1), 191. Available from: 10.1038/s41426-018-0194-y 30482898 PMC6258732

[mve12779-bib-0078] Peyton, E.L. , & Harrison, B.A. (1979) *Anopheles* (Cellia) *dirus*, a new species of the Leucosphyrus Group from Thailand (Diptera: Culicidae). Mosquito Systematics, 11, 40–52.

[mve12779-bib-0048] Poinsignon, A. , Samb, B. , Doucoure, S. , Drame, P.M. , Sarr, J.B. , Sow, C. et al. (2010) First attempt to validate the gSG6‐P1 salivary peptide as an immuno‐epidemiological tool for evaluating human exposure to *Anopheles funestus* bites. Tropical Medicine & International Health, 15(10), 1198–1203. Available from: 10.1111/j.1365-3156.2010.02611.x 20723184

[mve12779-bib-0049] Ponlawat, A. & Harrington, L. (2005) Blood feeding patterns of *Aedes aegypti* and *Aedes albopictus* in Thailand. Journal of Medical Entomology, 42(5), 844–849. 16363170 10.1093/jmedent/42.5.844

[mve12779-bib-0050] Remoue, F. , Cisse, B. , Ba, F. , Sokhna, C. , Herve, J.P. , Boulanger, D. et al. (2006) Evaluation of the antibody response to *anopheles* salivary antigens as a potential marker of risk of malaria. Transactions of the Royal Society of Tropical Medicine and Hygiene, 100(4), 363–370. Available from: 10.1016/j.trstmh.2005.06.032 16310235

[mve12779-bib-0051] Ribeiro, J.M. (2000) Blood‐feeding in mosquitoes: probing time and salivary gland anti‐haemostatic activities in representatives of three genera (*Aedes, anopheles, Culex*). Medical and Veterinary Entomology, 14, 142–148. 10872858 10.1046/j.1365-2915.2000.00227.x

[mve12779-bib-0052] Ribeiro, J.M. , Mans, B.J. & Arca, B. (2010) An insight into the sialome of blood‐feeding Nematocera. Insect Biochemistry and Molecular Biology, 40(11), 767–784. Available from: 10.1016/j.ibmb.2010.08.002 20728537 PMC2950210

[mve12779-bib-0053] Richards, S. , Ponnusamy, L. , Unnasch, T. , Hassan, H. & Apperson, C. (2006) Host‐feeding patterns of *Aedes albopictus* (Diptera: Culicidae) in relation to availability of human and domestic animals in suburban landscapes of Central North Carolina. Journal of Medical Entomology, 43(3), 543–551. 16739414 10.1603/0022-2585(2006)43[543:hpoaad]2.0.co;2 PMC2577020

[mve12779-bib-0054] Rosa‐Silva, H. , Cardoso, J.G. , Reis‐Júnior, R. , Corgosinho, P.H.C. , Faria, M.L. , Ribeiro, S.P. et al. (2023) Coexistence and spatial distribution of invasive and sylvatic container‐breeding mosquitoes in City–Forest ecotone within the Brazilian semi‐arid. Diversity, 15(7), 822. Available from: 10.3390/d15070822

[mve12779-bib-0055] Saeung, A. (2012) *Anopheles* (Diptera: Culicidae) species complex in Thailand: identification, distribution, bionomics and malaria‐vector importance. International Journal of Parasitology Research, 4(1), 75–82.

[mve12779-bib-0056] Sagna, A.B. , Yobo, M.C. , Elanga Ndille, E. & Remoue, F. (2018) New Immuno‐epidemiological biomarker of human exposure to *Aedes* vector bites: from concept to applications. Tropical Medicine and Infectious Disease, 3(3), 80. Available from: 10.3390/tropicalmed3030080 30274476 PMC6161005

[mve12779-bib-0057] Sahavechaphan, N. , Chatrattikorn, A. , Sadakorn, P. , Areechokechia, D. & Iamsirithaworn, S. (2020) Demystifying essential containers and places for *Aedes* Mosquito control in Thailand. Research Square. 1–17. Available from: 10.21203/rs.3.rs-123245/v1

[mve12779-bib-0079] Sallum, M.A.M. , Peyton, E.L. , & Wilkerson, R.C. (2005) Six new species of the *Anopheles leucosphyrus* group, reinterpretation of *An. elegans* and vector implications. Medical and Veterinary Entomology, 19(2), 158–199. Portico. Available from: 10.1111/j.0269-283x.2005.00551.x 15958025

[mve12779-bib-0058] Schmid, M.A. , Kauffman, E. , Payne, A. , Harris, E. & Kramer, L.D. (2017) Preparation of mosquito salivary gland extract and intradermal inoculation of mice. Bio‐Protocol, 7(14), e2407. Available from: 10.21769/BioProtoc.2407 28932759 PMC5602574

[mve12779-bib-0059] Schneider, B.S. & Higgs, S. (2008) The enhancement of arbovirus transmission and disease by mosquito saliva is associated with modulation of the host immune response. Transactions of the Royal Society of Tropical Medicine and Hygiene, 102(5), 400–408. Available from: 10.1016/j.trstmh.2008.01.024 18342898 PMC2561286

[mve12779-bib-0060] Service, M . (1993) Mosquito ecology: field sampling methods. Amsterdam, Netherlands: Elsevier Science.

[mve12779-bib-0061] Silver, J. (2008) Mosquito ecology field sampling methods, Third Edition edition. New York City, United State: Springer.

[mve12779-bib-0062] Srisuka, W. , Sulin, C. , Sommitr, W. , Rattanarithikul, R. , Aupalee, K. , Saeung, A. et al. (2022) Mosquito (Diptera: Culicidae) diversity and community structure in Doi Inthanon National Park, northern Thailand. Insects, 13(9), 814. Available from: 10.3390/insects13090814 36135515 PMC9505505

[mve12779-bib-0063] Thai National Parks . (2021) Welcome to Thai National Parks. Prachuap Khiri Khan: GibbonWoot. Available from: https://www.thainationalparks.com.

[mve12779-bib-0064] Thammapalo, S. , Moonmek, S. , Prikchoo, P. & Pengsakul, T. (2021) The potential container habitats of chikungunya vector in outbreak area of southern Thailand. Journal of the American Mosquito Control Association, 37(3), 4–160. 10.2987/20-6965.1 34407170

[mve12779-bib-0065] Thongsripong, P. , Green, A. , Kittayapong, P. , Kapan, D. , Wilcox, B. & Bennett, S. (2013) Mosquito vector diversity across habitats in central Thailand endemic for dengue and other arthropod‐borne diseases. PLoS Neglected Tropical Diseases, 7(10), e2507. Available from: 10.1371/journal.pntd.0002507 24205420 PMC3814347

[mve12779-bib-0066] Tongthainan, D. , Mongkol, N. , Jiamsomboon, K. , Suthisawat, S. , Sanyathitiseree, P. , Sukmak, M. et al. (2020) Seroprevalence of dengue, Zika, and chikungunya viruses in wild monkeys in Thailand. The American Journal of Tropical Medicine and Hygiene, 103(3), 1228–1233. Available from: 10.4269/ajtmh.20-0057 32588813 PMC7470562

[mve12779-bib-0067] Treesucon, U. (1988) A survey of stump‐tailed macaques (*Macaca arctoides*) in Thailand. Natural History Bulletin of the Siam Society, 36, 61–70.

[mve12779-bib-0068] Trevejo, R. & Reeves, W. (2005) Antibody response to *Culex tarsalis* salivary gland antigens among sentinel chickens in California. The American Journal of Tropical Medicine and Hygiene, 72(4), 481–487. 15827292

[mve12779-bib-0069] Valentine, M.J. , Murdock, C.C. & Kelly, P.J. (2019) Sylvatic cycles of arboviruses in non‐human primates. Parasites & Vectors, 12(1), 463. Available from: 10.1186/s13071-019-3732-0 31578140 PMC6775655

[mve12779-bib-0070] Waitayakul, A. , Somsri, S. , Sattabongkot, J. , Looareesuwan, S. , Cui, L. & Udomsangpetch, R. (2006) Natural human humoral response to salivary gland proteins of *anopheles* mosquitoes in Thailand. Acta Tropica, 98(1), 66–73. Available from: 10.1016/j.actatropica.2006.02.004 16530153

[mve12779-bib-0071] Washino, R.K. (1983) Mosquito host Bloodmeal identification: methodology and data analysis. Annual Review of Entomology, 28, 179–201. 10.1146/annurev.en.28.010183.001143 6131641

[mve12779-bib-0072] Wongkamchai, S. , Khongtak, P. , Leemingsawat, S. , Komalamisra, N. , Junsong, N. , Kulthanan, K. et al. (2010) Comparative identification of protein profiles and major allergens of saliva, salivary gland and whole body extracts of mosquito species in Thailand. Asia‐Pacific Journal of Public Health, 28, 162–169. 21038786

[mve12779-bib-0073] World Health Organization . (2020) Zoonoses. https://www.who.int/news-room/fact-sheets/detail/zoonoses.

[mve12779-bib-0074] Yanmanee, S. , Seethamchai, S. , Kuamsab, N. , Karaphan, S. , Suwonkerd, W. , Jongwutiwes, S. et al. (2023) Natural vectors of *plasmodium knowlesi* and other primate, avian and ungulate malaria parasites in Narathiwat Province, southern Thailand. Scientific Reports, 13(1), 8875. Available from: 10.1038/s41598-023-36017-3 37264067 PMC10235068

[mve12779-bib-0075] Yobo, C.M. , Sadia‐Kacou, C.A.M. , Adja, M.A. , Elanga‐Ndille, E. , Sagna, A.B. , Guindo‐Coulibaly, N. et al. (2018) Evaluation of human exposure to *Aedes* bites in rubber and palm cultivations using an Immunoepidemiological biomarker. BioMed Research International, 2018, 3572696. Available from: 10.1155/2018/3572696 30175128 PMC6106716

