# Supplementary material for: Prevalence of culicine salivary antibodies in non‐human primates living in national parks in Thailand
Source: Med Vet Entomol. 2024 Nov 25;39(2):278–90. doi: 10.1111/mve.12779 (PMC12054348; doi:10.1111/mve.12779)
Supplement: Supplementary file 1 — Supplementary Information S1. Table S1. Mean anti‐mosquito (Aedes aegypti, Ae. albopictus and Culex quinquefasciatus) SGE (salivary gland extract) antibody endpoint titres (Log2) of Macaca arctoides, M. leonina and M. fascicularis in Thailand. Table S2. Geometric mean anti‐mosquito (Aedes aegypti, Ae. albopictus and Culex quinquefasciatus) SGE (salivary gland extract) antibody endpoint titres (Log2) of Macaca arctoides, M. leonina and M. fascicularis in Thailand. [file MVE-39-278-s001.docx]

**Supplementary Information 1**

**Table 1. Mean anti-mosquito (*Aedes aegypti, Ae. albopictus,* and *Culex quinquefasciatus*) SGE (salivary gland extract) antibody endpoint titers (Log_2_) of *Macaca arctoides, M. leonina,* and *M. fascicularis* in Thailand**

| **Species** | **Kaeng Krachan National Park**  **(*M. arctoides*)** | **Khao Yai National Park**  **(*M. leonina*)** | **Mu Ko Ranong National Park**  **(*M. fascicularis*)** |
| --- | --- | --- | --- |
| *Ae. aegypti* | 19,491 (13,726-20,609) | 22,678 (9,483-21,989) | 41,600 (12,016-109,084) |
| *Ae. albopictus* | 46,158 (22,708-42,120) | 32,835 (7,333-22,344) | 28,800 (10,402-63,001) |
| *Cx. quinquefasciatus* | 30,642 (21,636-31,590) | 14,545 (10,147-16,147) | 25,600 (25,600-25,600) |

**Table 2. Geometric mean anti-mosquito (*Aedes aegypti, Ae. albopictus,* and *Culex quinquefasciatus*) SGE (salivary gland extract) antibody endpoint titers (Log_2_) of *Macaca arctoides, M. leonina,* and *M. fascicularis* in Thailand**

| **Species** | **Kaeng Krachan National Park**  **(*M. arctoides*)** | **Kaeng Krachan National Park**  **(*M. leonina*)** | **Kaeng Krachan National Park**  **(*M. fascicularis*)** |
| --- | --- | --- | --- |
| *Ae. aegypti* | 16,819 (13,726-20,609) | 14,440 (9,483-21,989) | 36,204 (12,016-109,084) |
| *Ae. albopictus* | 30,927 (22,708-42,120) | 12,800 (7,333-22,344) | 25,600 (10,402-63,001) |
| *Cx. quinquefasciatus* | 26,143 (21,636-31,590) | 12,800 (10,147-16,147) | 25,600 (25,600-25,600) |
